# Effect of Hydrothermal Coatings of Magnesium AZ31 Alloy on Osteogenic Differentiation of hMSCs: From Gene to Protein Analysis

**DOI:** 10.3390/ma18061254

**Published:** 2025-03-12

**Authors:** Viviana Costa, Lavinia Raimondi, Simone Dario Scilabra, Margot Lo Pinto, Daniele Bellavia, Angela De Luca, Pasquale Guglielmi, Angela Cusanno, Luca Cattini, Lia Pulsatelli, Matteo Pavarini, Roberto Chiesa, Gianluca Giavaresi

**Affiliations:** 1 CS-Surgical Sciences and Technologies-SS Omics Science Platform for Personalized Orthopedics, IRCCS Istituto Ortopedico Rizzoli, 40136 Bologna, Italy; viviana.costa@ior.it (V.C.); daniele.bellavia@ior.it (D.B.); angela.deluca@ior.it (A.D.L.); gianluca.giavaresi@ior.it (G.G.); 2 Ri.MED Foundation, IRCCS ISMETT, Via Ernesto Tricomi 5, 90145 Palermo, Italy; sdscilabra@fondazionerimed.com (S.D.S.); mlopinto@fondazionerimed.com (M.L.P.); 3 Department of Mechanics, Mathematics and Management, Polytechnic University of Bari, 70125 Bari, Italy; pasquale.guglielmi@poliba.it (P.G.); angela.cusanno@poliba.it (A.C.); 4 Laboratory of Immunorheumatology and Tissue Regeneration, IRCCS Istituto Ortopedico Rizzoli, Via di Barbiano 1/10, 40136 Bologna, Italy; luca.cattini@ior.it (L.C.); lia.pulsatelli@ior.it (L.P.); 5 Department of Chemistry, Materials and Chemical Engineering ‘G. Natta’, Politecnico di Milano, 20135 Milan, Italy; matteo.pavarini@polimi.it (M.P.); roberto.chiesa@polimi.it (R.C.)

**Keywords:** Mg alloys, osteointegration, bone regeneration, soluble factors release

## Abstract

An Mg-based alloy device manufactured via a superplastic forming process (Mg-AZ31+SPF) and coated using a hydrothermal method (Mg AZ31+SPF+HT) was investigated as a method to increase mechanical and osteointegration capability. The cell viability and osteointegrative properties of alloy-derived Mg AZ31+SPF and Mg AZ31+SPF+HT extracts were investigated regarding their effect on human mesenchymal stem cells (hMSCs) (maintained in basal (BM) and osteogenic medium (OM)) after 7 and 14 days of treatment. The viability was analyzed through metabolic activity and double-strand DNA quantification, while the osteoinductive effects were evaluated through qRT-PCR, osteoimage, and BioPlex investigations. Finally, a preliminary liquid mass spectrometry analysis was conducted on the secretome of hMSCs. Biocompatibility analysis revealed no toxic effect on cells’ viability or proliferation during the experimental period. A modulation effect was observed on the osteoblast pre-commitment genes of hMSCs treated with Mg-AZ31+SPF+HT in OM, which was supported by mineralization nodule analysis. A preliminary mass spectrometry investigation highlighted the modulation of protein clusters involved in extracellular exosomes, Hippo, and the lipid metabolism process. In conclusion, our results revealed that the Mg AZ31+SPF+HT extracts can modulate the canonical and non-canonical osteogenic process in vitro, suggesting their possible application in bone tissue engineering.

## 1. Introduction

Bone regeneration involves a complex mechanism regulated by a balance between the micro- and macro-environments. This balance is altered in skeletal diseases, tumors, lesions, and immunodeficiencies, activating cell proliferation and differentiation in the bone niche [1,2,3].

Various aspects of the regeneration process have been studied, including hypoxia, angiogenesis, exosome release, the role of miRNAs and lncRNAs, and IL secretion, to discover a suitable biomaterial for clinical use that does not disrupt the signaling involved in bone regeneration [4,5].

The biocompatibility and osteointegrative ability of many orthopaedic biomaterials, such as steel, chromium–cobalt alloys, and titanium alloys, are well known, along with their functional limitations. Among resorbable metals and their alloys, magnesium (Mg) could be considered the most interesting due to its biocompatibility, if not for its high corrosion rate [6,7,8,9]. A number of criteria must be met by medical materials. Implants must be designed to mimic and support bone structure and bone tissue formation (osteogenesis) and to do so in a morphologically appropriate way [10]. Magnesium represents the primary macroelement of the human body and has been demonstrated to significantly enhance bone formation and regeneration [11,12]. Magnesium might be a useful material for implants due to its biocompatibility and osteogenic and angiogenic potential, but the rapid degradation of magnesium implants in situ represents a significant limitation [10,13,14]. The high rate of corrosion, even if the material itself is non-toxic, is a critical issue in terms of the rapid loss of mechanical integrity and can result in osteolysis, which causes the degeneration and weakening of the bones and the loss of the mechanical integration of the implants, increasing the risk factor for implant failure [9,15,16]. Consequently, this increases the levels of hydrogen gas and promotes the activation of the inflammatory process at the implant site [5]. It has been observed that the presence of biological fluids near the implant and exposure to pH level fluctuations accelerates the deterioration of Mg, causing damage to the surrounding area. As a result, this has been shown to disrupt the delicate balance between pro-fibrotic and anti-fibrotic factors, including procollagen type I alpha1. Tumor necrosis factor (TNF) alpha and anti-fibrotic factors, including interferon (IFN) gamma and transforming growth factor (TGF)-β1 and -β2, influence the expression of signaling pathways involved in bone regeneration, such as Hippo signaling, lipid metabolism, and extracellular exosomes [4,9,17,18,19,20].

Treatments and surface coating represent effective methods to refine Mg alloys in order to boost corrosion resistance and improve the long-term efficacy of Mg-based implants overall [21,22,23]. In recent years, research has concentrated on developing alternative methods to enhance the coating efficiency of Mg alloys, which has been identified as a key challenge in this field. Tatullo et al. demonstrated the merits of devices fabricated using Mg alloys; produced using the superplastic forming (SPF) technique, which facilitates the fabrication of complex and customised geometries; and subsequently subjected to coating with hydrothermal treatment (HT) [24]. In this context, hydrothermal methods represent a promising solution for surface modification, as they enable the formation of continuous and compact magnesium hydroxide (Mg(OH)_2_)-based layers. These layers have the capacity to act as an effective barrier against corrosion-promoting physiological media while exhibiting a generally well-tolerated chemical nature [25,26]. Furthermore, this technique boasts numerous significant advantages that position it as a notable contender in the array of surface modification techniques currently under investigation. These advantages include its foundation on a remarkably uncomplicated, resilient, cost-effective, and environmentally friendly process, in addition to its capacity to yield coatings of considerable thickness (tens of micrometers) and remarkable adhesion (resulting from substrate conversion). In this study, an optimized hydrothermal process was employed with medium composition, time, and temperature conditions for the target substrate being experimentally tuned based on the established literature data [25,26,27,28]. This approach was undertaken to ensure the synthesis of uniform, dense, and protective coatings with the potential for high biocompatibility and the promotion of osteogenesis.

The aim of this study was to investigate the effects of hydrothermal treatment on the Mg AZ31 alloy formed by SPF (Mg AZ31+SPF+HT) in terms of cell viability and osseointegration. To this end, an in vitro model was developed in which hMSCs were treated with extracts obtained from the Mg AZ31+SPF+HT alloy, as previously described by De Luca et al. [29] The response of hMSCs to treatment with Mg AZ31+SPF+HT extracts was analyzed in terms of protein secretion over time. The release of fibrotic and anti-fibrotic proteins was evaluated because Mg implants are traditionally attributed with anti-inflammatory properties, but an initial inflammatory phase is usually observed, which can induce fibrosis. However, when Mg implants are successfully integrated into the bone implantation site, these phenomena fully subside [30]. Mass spectrometry analysis of the secretome was conducted to highlight any potential enrichment of proteins involved in critical requirements during osteogenesis, from the main demands of cellular energy metabolism to the activation of crucial signaling pathways for bone development and homeostasis. Overall, the results obtained highlighted the effectiveness of hydrothermal coating treatment in promoting the molecular mechanisms underlying the osseointegration process.

## 2. Materials and Methods

### 2.1. Cell Lines

Human MSCs were cultured in mesenchymal stem cell growth medium (MSCGM™ Bullet Kit, Lonza, Walkersville, MD, USA) to expand the cells without inducing differentiation. The culture medium was changed every 3 days, and the cells were split at 80–90% of confluence using StemPro Accutase (Gibco by Life Technologies, Grand Islands, NY, USA). To perform osteogenic differentiation, the hMSCs were treated with hMSC mesenchymal stem cell osteogenic differentiation medium (OM) (hMSC Osteogenic Differentiation Bullet Kit™, Lonza). For the in vitro treatments, the hMSCs were seeded onto 12-well plates at a concentration of 25.000 cells/well in the presence of extracts derived from Mg alloys, obtained as described by Tatullo M. et al. [24] and using extract dilutions 25% PBS with 75% DMEM, as suggested by De Luca A. et al. [29]. After 7 and 14 days of culture, samples were recovered and texted. Briefly, cylindrical Mg AZ31 alloy samples (10 mm diameter, 1 mm thick) were fabricated via superplastic forming (SPF) [31]. The samples were then degreased with acetone, etched for 10 sec in a solution of 4 M acetic acid and 1 M nitric acid (Sigma-Aldrich, Merck, Darmstadt, Germany) to remove surface contaminants, and rinsed thoroughly in Milli-Q water for 5 min using an ultrasonic bath (Elmasonic S60, Elma Ultrasonic, Singen, Germany). The samples were then placed in a sealed, Teflon-lined stainless-steel hydrothermal synthesis reactor, filled up to 70% with de-ionized water, at 160 °C for 4 h, producing a uniform magnesium hydroxide (Mg(OH)_2_)-based coating (≈20 μm thick). Following a final ultrasonic cleaning in Milli-Q water and drying in air, each implant was placed in a PA/PE double pouch, thermo-sealed, and gamma-sterilized at 25.0 kGy. The continuity and uniformity of the hydrothermal coatings were verified by surface and cross-sectional SEM imaging performed on randomly selected benchmark samples during production.

### 2.2. hMSC Viability (WST-1 Test)

WST-l colorimetric reagent (Roche Diagnostics GmbH, Manheim, Germany) was used to evaluate cell viability. Briefly, WST-1 reagent (10% vol/vol) was added to the cell monolayer in each well. After 4 h of incubation, the formazan dye produced by viable cells was quantified spectrophotometrically at 450 nm by Bio-Rad Microplate Reader (Bio-Rad Laboratories, Hercules, CA, USA), and the results were reported as the percentage of viable cells compared to the untreated group.

### 2.3. DNAds Concentration (PicoGreen Assay)

The concentration of dsDNA content was quantified by using a fluorimetric Quant-iT PicoGreen dsDNA Assay Kit (Invitrogen™, Life Technologies—EuroClone S.p.A, Pero-Milan, Italy). After the samples were washed with phosphate-buffered saline, 250 μL of lysis solution was added to each well plate, and cell lysis was then completed via 3 freeze–thaw cycles. After 5 min of incubation at room temperature (RT) while protected from light, DNAds content of the lysates was calculated by adding 100 μL of fluorescent nucleic acid stain to each scaffold. Fluorescence was measured using a GloMax multiwell plate reader (GloMax, Promega Corporation, Madison, WI, USA). Data are reported as the fold of induction (FOI) between the treated and untreated cells (n = 3 duplicates).

### 2.4. RNA Extraction and Real-Time PCR

Total RNA was extracted using the commercially available illustraRNAspin Mini Isolation Kit (GE Healthcare, Milan, Italy), following the manufacturer’s instructions. RNA was reverse transcribed to cDNA using the HighCapacity cDNA Reverse Transcription Kit (Applied Biosystems, Fisher Scientific Italia, Segrate, Italy), and relative real-time PCR was performed in duplicate for each data point using QuantiTect Primers (Qiagen Srl, Milan, Italy) (Table 1). Changes in the target mRNA content relative to the housekeeping gene β-actin were determined using the 2^−ΔΔCt^ method [32].

### 2.5. OsteoImage Mineralization Assay

The OsteoImage Mineralization Assay (PA-1503, Lonza) is a rapid, fluorescent in vitro assay for assessing bone cell mineralization. Cells were seeded in 12 wells (n = 3) at a concentration of 25.000 cells/well in basal and osteogenic medium with or without Mg extracts. At the end of the experimental period, either 7 or 14 days, the medium was removed and the cells washed with PBS w/o calcium or magnesium. After, the cells were fixed with methanol for 20 min and washed in PBS. OsteoImage™ Staining Reagent was added to the cells for 30 min at 4 °C protect them from light. Finally, the cells were washed in PBS 3 times and the fluorescence of the samples was observed using a NIKON fluorescence microscope at a grading of 10×.

### 2.6. Evaluation of Supernatant Soluble Factors

The concentrations of soluble factors were evaluated by multiplex bead immunoassay. Samples were assayed in duplicate, and the concentrations of procollagen type I alpha1, tumor necrosis factor (TNF) alpha, interferon (IFN) gamma, and transforming growth factor (TGF)-β1 and -β2 were simultaneously evaluated using commercially available bead-based sandwich immunoassay kits (Luminex performance Assay multiplex kits, R&D Systems, Minneapolis, MN, USA), following the manufacturer’s instructions. Briefly, distinct sets of fluorescently dyed beads loaded with capture monoclonal antibodies specific for each factor to be tested were used. Samples (50 µL/well) or standards (50 µL/well) were incubated with 50 µL of pre-mixed bead sets in the wells of a 96-well microtiter plate. After incubation and washing, 50 µL of biotin–antibody mixture was added for one hour, and then the samples were washed and resuspended with 50 µL of fluorescent streptavidin for 30 min. The immunocomplexes formed on distinct beads were quantified using the Bio-Plex Protein Array System (Bio-Rad Laboratories, Hercules, CA, USA). Data were analyzed using the Bio-Plex Manager software version 6.0 (Bio-Rad Laboratories, Hercules, CA, USA). In general, at least six standards were accepted and used to establish standard curves following a five-parameter logistic regression model (5 PL). Sample concentrations were immediately interpolated from the standard curves.

### 2.7. Conditional Medium Preparation

A measure of 10 μg of conditioned media (secretome) was collected and subjected to proteolytic digestion using a filter-assisted sample preparation (FASP) protocol with 10 kDa Vivacon spin filters (Sartorius, Göttingen, Germany) [33].

Briefly, proteins were reduced by the addition of 1 M Dithiothreitol (DTT, Thermo Fischer Scientific) in 100 mM Tris/HCl containing 8 M urea, pH 8.5, for 30 min at 37 °C. Proteins were then alkylated in 50 mM iodoacetamide (IAA, Thermo Fischer Scientific) for 5 min at room temperature and washed twice in 100 mM Tris/HCl containing 8 M urea, pH 8.0, at 14,000 × g for 30 min. Proteins were digested with 0.2 μg LysC (Promega, Madison, WI, USA) in 25 mM Tris/HCl containing 2 M urea, pH 8.0, overnight and with 0.1 μg trypsin (Promega) in 50 mM ammonium bicarbonate for 4 h. The resulting peptides were desalted by stop-and-go extraction (STAGE) on reverse-phase C18 (Supelco Analytical Products, part of Sigma-Aldrich, Bellefonte, PA, USA) and eluted in 40 μL of 60% acetonitrile in 0.1% formic acid [34]. Then, the volume was reduced in a SpeedVac (Thermo Fisher Scientific, Waltham, MA, USA) and the peptides resuspended in 20 μL of 0.1% formic acid. The peptide’s concentration was measured by a NanoDrop microvolume spectrophotometer (Thermo Fischer Scientific), and LC-MS/MS analysis was conducted on 1 μg of peptide.

### 2.8. LC-MS/MS

To achieve high sensitivity, a nanoLC system (Vanquish Neo UHPLC—part of Thermo Scientific) using an Acclaim PEPMap C18 column (25 cm × 75 µm ID, Thermo Scientific, Waltham, MA, USA) was coupled online to an Exploris 480 mass spectrometer (Thermo Fischer Scientific). Peptides were separated using a 130 min binary gradient of water and acetonitrile containing 0.1% formic acid [33]. Data-independent acquisition (DIA) was performed using a full MS1 scan (400 *m*/*z* to 1200 *m*/*z*) followed by 60 sequential DIA windows with an overlap of 1 *m*/*z* and the window placement optimization option enabled. Full scans were acquired with a resolution of 120,000, an automatic gain control (AGC) of 3 × 10^6^, and a maximum injection time of 50 ms. Afterwards, 60 isolation windows were scanned with a resolution of 30,000 and an AGC of 8 × 10^5^, while maximum injection time was set to auto to achieve the optimal cycle time. Collision-induced dissociation fragmentation was induced with 30% of the normalized HCD collision energy [34].

The data were analyzed by the software DIA-NN (version 1.8.1) using a predicted library generated from the in silico digested human Uniprot reference database, involving cuts at K* and R*, with two missed cleavages allowed, and with the minimal peptide length set at 6, which consisted of 20,923 proteins, 31,382 protein groups, and 6,894,911 precursors in 2,140,232 elution groups [35]. The false discovery rate for peptide and protein identification was set at 0.01%. Label-free quantification (LFQ) was used for protein quantification [36].

### 2.9. Statistical Analysis

Statistical analysis was conducted by using GraphPad Prism v.9. Data are reported as mean ± SD at a significance level of *p* < 0.05. After having verified the normal distribution and homogeneity of variance, one-way ANOVA was used followed by Tukey’s multiple comparisons test to compare data among two groups or more.

The functional classification of increased proteins from hMSC+Mg AZ31+SPF+HT extracts vs. hMSC+Mg AZ31+SPF extracts (7 and 14 days) was carried out using the Protein Analysis THrough Evolutionary Relationships (PANTHER) database (www.pantherdb.org, accessed on 8 November 2024), designed to classify proteins (and their genes). The PANTHER classification system combines genomes, gene function classifications, pathways, and statistical analysis tools to enable biologists to analyze big experimental data. The current system (PANTHER v.19.0) includes 144 complete genomes organized into gene families and subfamilies, annotated with Gene Ontology terms and sequences mapped to PANTHER pathways [37,38,39]. Protein–protein interactions were analyzed using the Search Tool for the Retrieval of Interacting Genes/Proteins (STRING; https://string-db.org, accessed on 23 January 2023) database of physical and functional interactions, specifically v11.5. Network nodes represent proteins and edges represent protein−protein associations. Protein clustering was conducted via K-means clustering (K = 3), also employing STRING.

## 3. Results

### 3.1. Cell Viability Response to Mg AZ31+SPF and Mg AZ31+SPF+HT Extract Treatments

**Figure 1:**
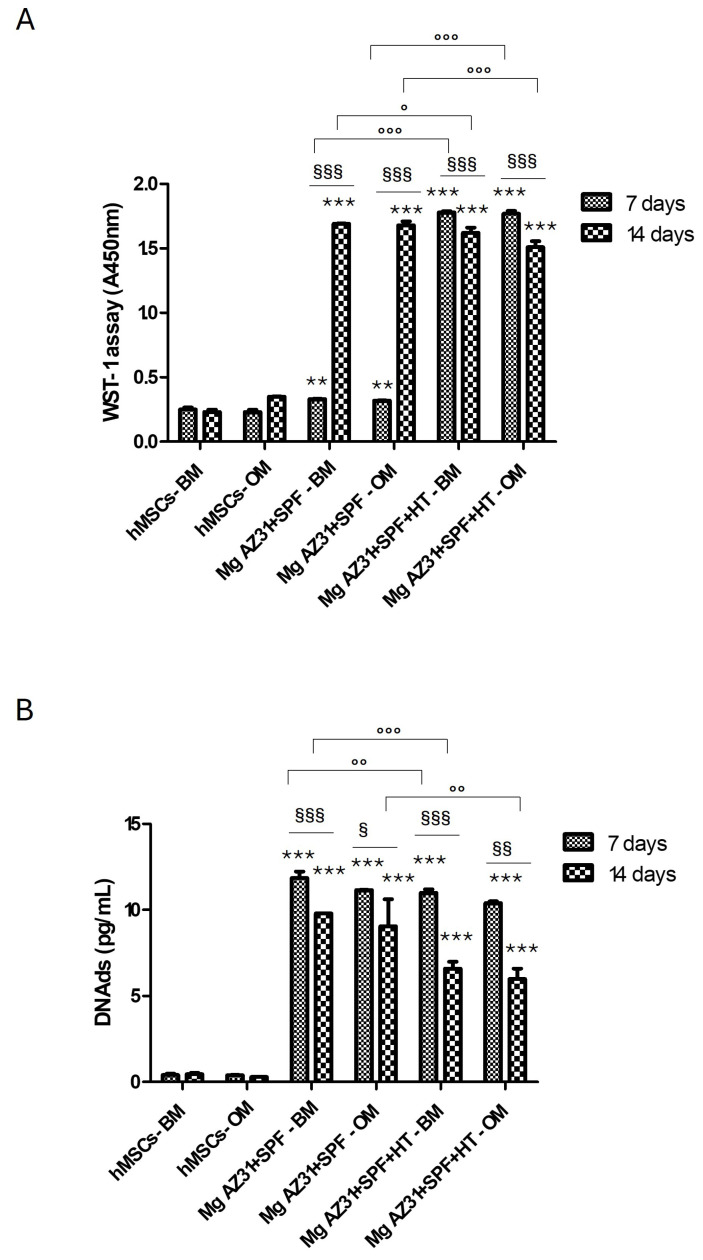
Viability evaluation of hMSCs treated with Mg AZ31 extracts. Cell viability assays of hMSCs treated for 7 and 14 days with Mg AZ31+SPF or Mg AZ31+SPF+HT extracts, maintained in BM or OM: WST-1 (**A**) and PicoGreen (**B**). Data are expressed as the value of a 450 nm or as the quantity of DNAds (pg/mL). A two-way ANOVA test was used to evaluate the effect of the different Mg AZ31 extract treatments in basal or osteogenic medium (°), for the same extracts over the experimental period (§), or compared to untreated cells (*) (one symbol, *p* < 0.05; two symbols, *p* < 0.005, and three symbols, *p* < 0.0005). All experiments were triplicated, with the data expressed as mean ± SD.

To evaluate hMSC viability after treatments with Mg extracts, the WST-1 assay and PicoGreen^®^ dsDNA quantification assay were used (Figure 1A,B). The metabolic activity of hMSCs maintained in medium both in presence of Mg AZ31+SPF+HT and Mg AZ31+SPF extracts was increased after 7 and 14 days of treatments compared to the untreated groups; no toxic effects were observed in treated samples during the experimental period.

Data were confirmed by the analysis of DNAds. An increase in dsDNA content was observed in hMSCs maintained in basal or osteogenic medium after 7 and 14 days of treatment with Mg AZ31+SPF or Mg AZ31+SPF+HT extracts compared to the untreated groups. During the experimental period, a significant decrease in DNAds on Mg AZ31+SPF+HT and Mg AZ31+SPF compared to the same samples at 7 days of treatments was revealed, although these samples still maintained a significant increase compared to the untreated cells.

### 3.2. Osteoinductive Effects of Mg AZ31+SPF and Mg AZ31+SPF+HT on hMSCs

**Figure 2:**
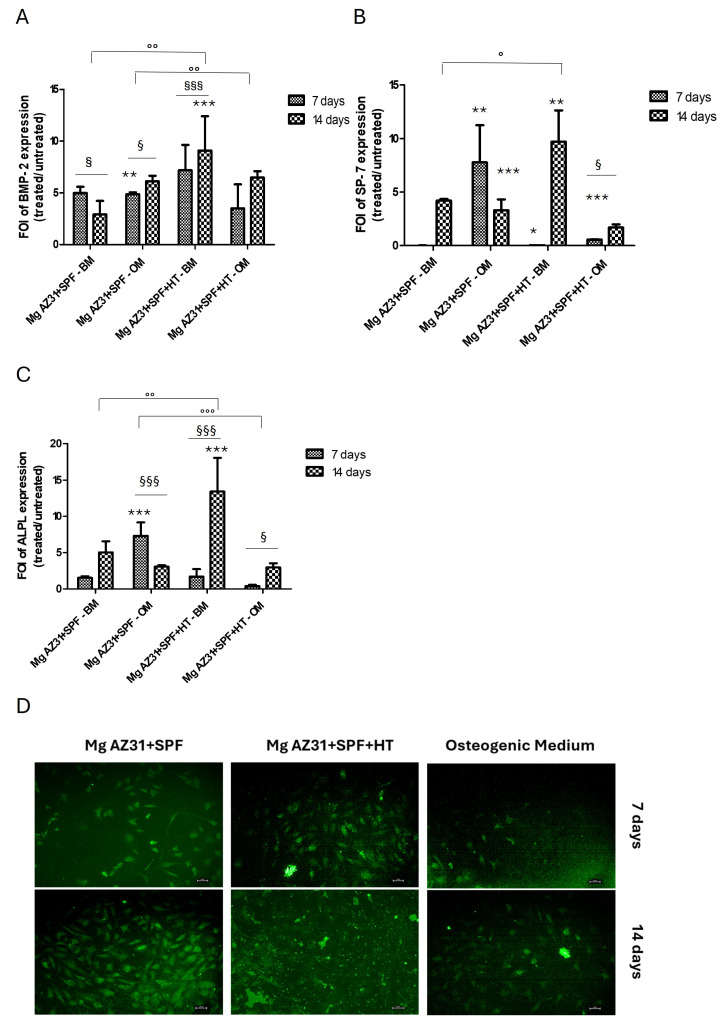
Osteogenic effects of Mg AZ31 extracts on hMSCs. qRT-PCR analysis of BMP-2 (**A**), SP-7 (**B**), and ALPL (**C**) gene expression on hMSCs treated for 7 and 14 days with Mg AZ31+SPF or Mg AZ31+SPF+HT extracts maintained in BM or OM. Data are expressed as fold of change (FOI) in gene expression (2^−ΔΔCt^) that occurred in treated vs. untreated cells. (**D**) OsteoImage mineralization assay conducted on hMSCs treated for 7 and 14 days with Mg AZ31+SPF or Mg AZ31+SPF+HT extracts maintained in OM. The images were acquired with 10× of grading on a Nikon Fluorescence Microscopy. A two-way ANOVA test was used to evaluate the effect of the different Mg AZ31 extract treatments in basal or osteogenic medium (°), for the same extracts over the experimental period (§), or compared to untreated cells (*) (one symbol, *p* < 0.05; two symbols, *p* < 0.005, and three symbols, *p* < 0.0005). All experiments were triplicated, with the data expressed as mean ± SD.

To investigate the osteogenic ability of hMSCs after 7 and 14 days of treatments with Mg AZ31+SPF or Mg AZ31+SPF+HT extracts, qRT-PCR analysis was performed. As shown in Figure 2A, the expression of BMP-2 increased in hMSCs maintained in BM after 14 days of culture. Conversely, hMSCs maintained in OM with Mg extracts showed a statistically significant increase in BMP-2 expression after 7 days. Expression analysis of SP7, an early osteogenic differentiation gene, showed increased expression compared to control cells in both media over the experimental period. Of particular interest is the modulation observed in OM cells treated with Mg AZ31+SPF+HT, which was statistically significant even between the two experimental time points (Figure 2B). Regarding ALPL, a significant modulation was observed after 14 days of treatments in hMSCs treated with Mg AZ31+SPF vs. Mg AZ31+SPF+HT (*p* < 0.0005) in BM, while in hMSCs maintained in OM, we observed an early increase in ALPL expression among the Mg AZ31+SPF+HT vs. Mg AZ31+SPF in OM (*p* < 0.0005) groups (Figure 2C). Based on the evidence obtained from the samples maintained in OM, we decided to evaluate the osteo-induction ability of Mg AZ31+SPF and Mg AZ31+SPF+HT extracts through OsteoImage mineralization assay (Figure 2D). The cells maintained in osteogenic medium for 7 and 14 days of treatments with Mg AZ31+SPF or Mg AZ31+SPF+HT extracts showed important mineralization nodule deposition on Mg AZ31+SPF+HT groups compared to others, especially after 14 days of culture.

### 3.3. Analysis of Fibrotic Potential of Mg AZ31+SPF and Mg AZ31+SPF+HT Extracts on hMSC Supernatant Soluble Factors

**Figure 3:**
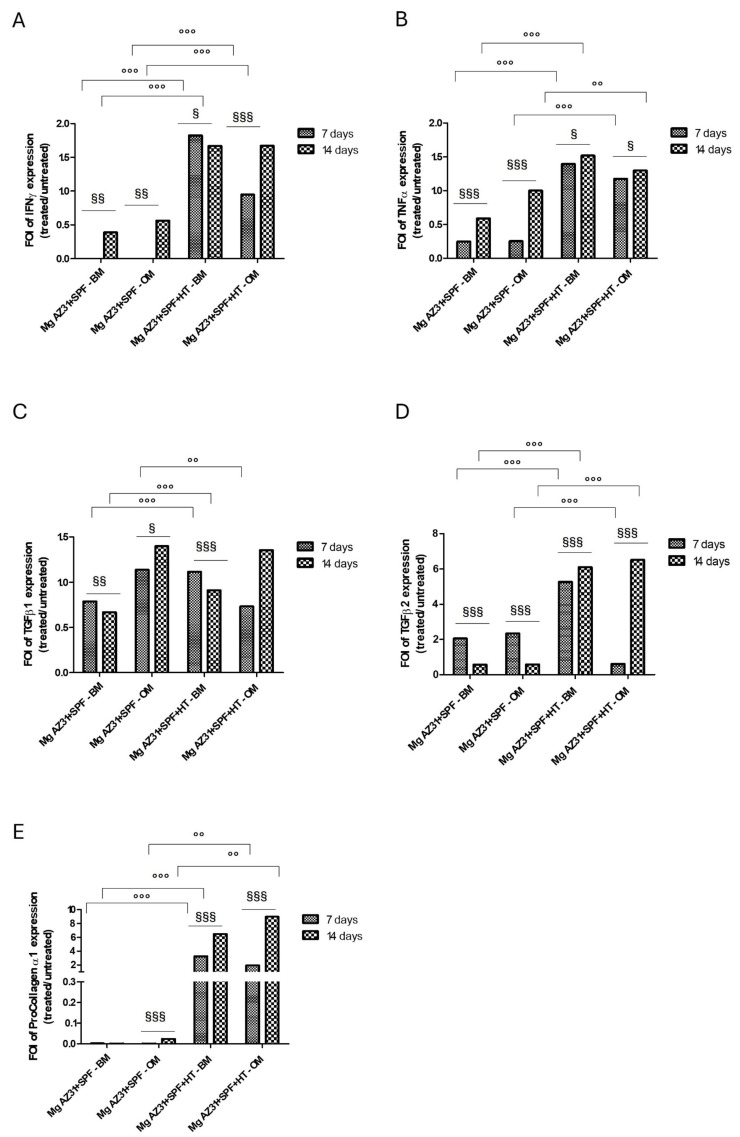
Analysis of fibrotic potential of Mg AZ31+SPF and Mg AZ31+SPF+HT extracts on hMSCs supernatant soluble factors. BioPlex analysis of the anti-fibrotic supernatant soluble factors (**A**) IFNγ and (**B**) TNFα and the pro-fibrotic soluble factors (**C**) TGFβ1, (**D**) TGFβ2, and (**E**) procollagen α1 released from hMSCs treated with Mg AZ31+SPF or Mg AZ31+SPF+HT extracts for 7 and 14 days in BM or OM. Data are expressed as the fold of induction (FOI) of treated vs. untreated cells. A two-way ANOVA test was used to evaluate the effect of the different Mg AZ31 extract treatments in basal or osteogenic medium (°) or for the same extracts over the experimental period (§) (one symbol, *p* < 0.05; two symbols, *p* < 0.005, and three symbols, *p* < 0.0005). All experiments were triplicated, with the data expressed as mean ± SD.

The characterization of the pro-fibrotic and anti-fibrotic factors involved in the osteointegration process was performed using a Bioplex approach. The supernatant recovered from hMSCs maintained in BM or OM medium after treatments with Mg AZ31+SPF or Mg AZ31+SPF+HT was evaluated. The data in Figure 3 show an increased release of the anti-fibrotic soluble factors IFN-γ and TNFα from hMSCs treated with Mg AZ31+SPF+HT extracts compared to those treated with Mg AZ31+SPF, in either culture medium and especially after 14 days of treatment. The amounts of pro-fibrotic factors released seem to increase in a relevant manner after treatments with Mg AZ31+SPF+HT compared to Mg AZ31+SPF extracts, especially for TGFβ-2 and procollagen 1α when hMSCs are maintained in OM for 14 days. These data support the beneficial effect of OM, which recreates the bone microenvironment in vitro, on the osteogenic activity of MgAZ31+SPF+HT.

### 3.4. Preliminary Mass Spectrometric Investigation of hMSCs Treated with Mg AZ31+SPF or Mg AZ31+SPF+HT Extracts

**Figure 4:**
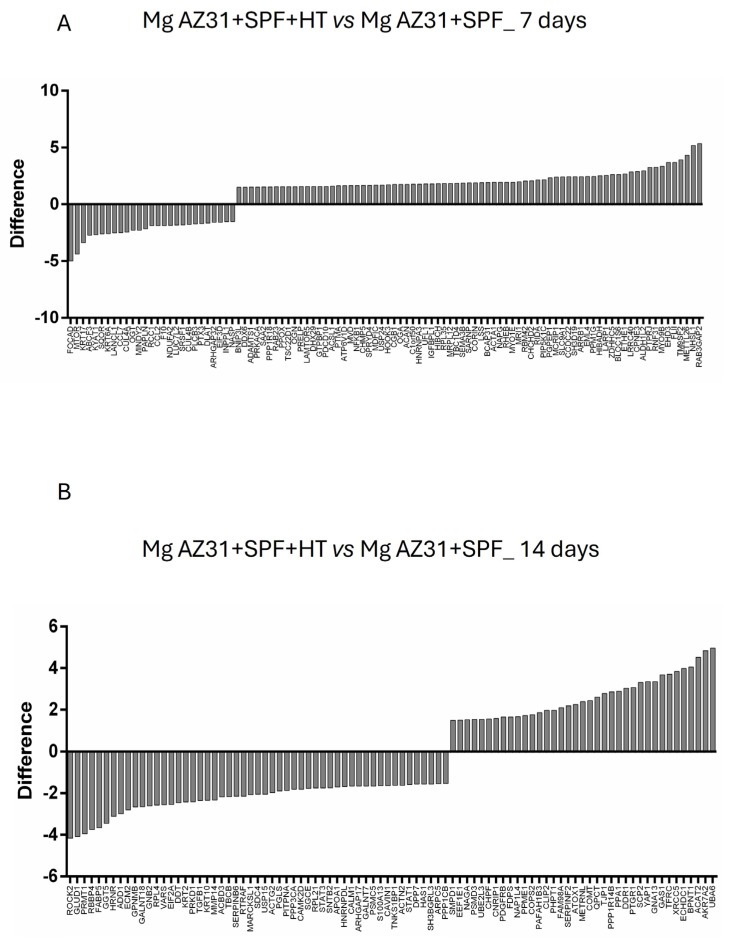
Mass spectrometric investigation of hMSCs treated with Mg AZ31+SPF or Mg AZ31+SPF+HT extracts. Representation of up- and downregulated proteins derived from the comparison of secretomes from hMSCs treated with Mg AZ31+SPF+HT extracts versus secretomes from hMSCs treated with Mg AZ31+SPF extracts, after 7 days (**A**) or 14 days (**B**). A fold of indication > 1.5 was used as the cut-off.

A preliminary analysis of the hMSC secretome was performed to determine how the tested material extracts could modulate the microenvironment in terms of protein release. A liquid chromatography with tandem mass spectrometry (LC-MS-MS) was used to analyze the protein profiles of secretome obtained from hMSCs cultured with Mg AZ31+SPF or Mg AZ31+SPF+HT extracts for 7 and 14 days in OM. The mass spectrometry analysis revealed a higher abundance of 75 proteins after 7 days of treatments when comparing Mg AZ31+SPF+HT vs. Mg AZ31+SPF and 37 proteins after 14 days of treatment (Figure 4A,B).

**Figure 5:**
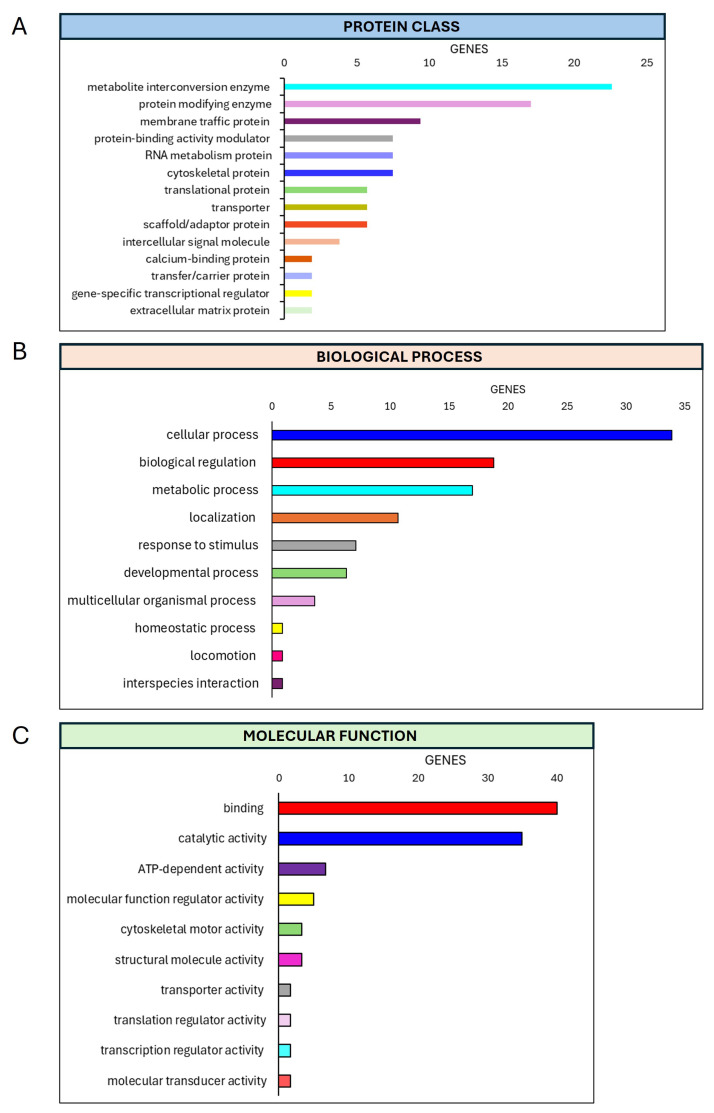
PHANTER analysis of secretome data. PANTHER pie charts of the (**A**) protein classes, (**B**) biological processes, and (**C**) molecular functions associated with increased proteins derived from the secretome of hMSCs treated with Mg AZ31+SPF+HT extracts versus the secretome of hMSCs treated with Mg AZ31+SPF extracts for 7 days.

**Figure 6:**
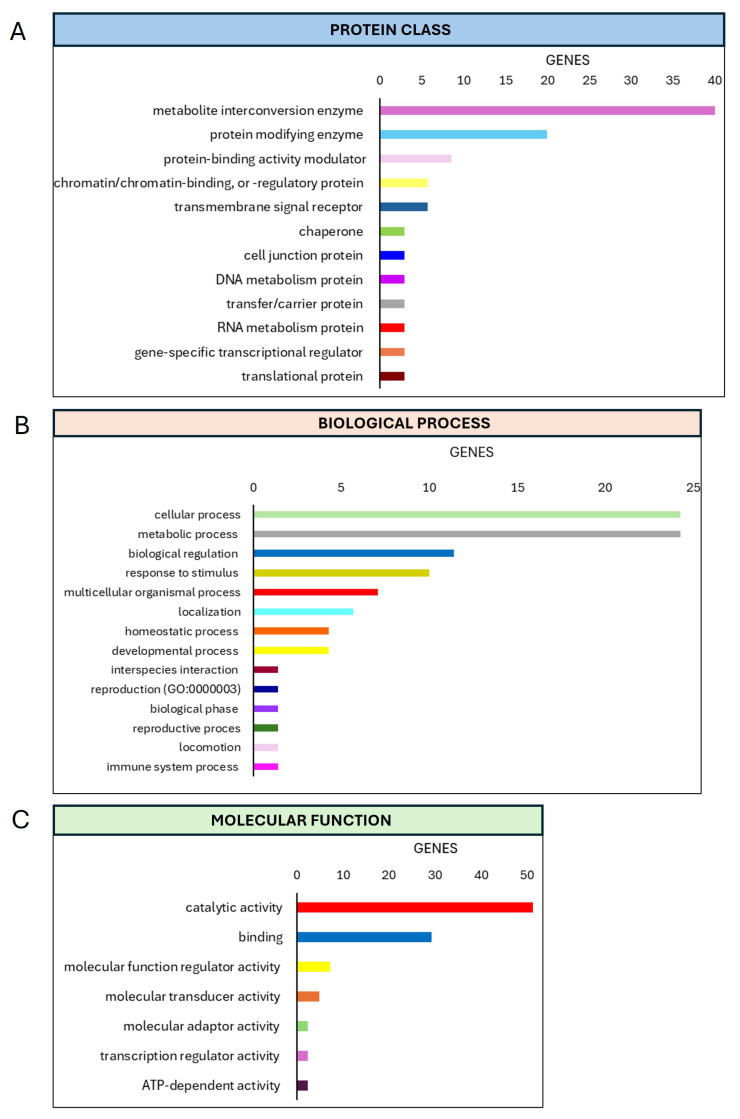
PHANTER analysis of secretome data. PANTHER pie charts of the (**A**) protein classes, (**B**) biological processes, and (**C**) molecular functions associated with increased proteins derived from the secretome of hMSCs treated with Mg AZ31+SPF+HT extracts versus the secretome of hMSCs treated with Mg AZ31+SPF extracts for 14 days.

The PANTHER analysis was performed for three different investigations: molecular function, protein class, and biological process (Figure 5 and Figure 6). The classification system used to identify proteins in order to facilitate high-throughput analysis of the secretome data revealed the following after 7 days of treatments: concerning the molecular function classification, a major expression was observed for the catalytic activity (35%) and binding (40%); regarding the protein class classification, metabolite interconversion enzyme (22.6%) and protein modify enzyme (17%) displayed relevant regulation; finally, regarding biological process classification, the proteins most expressed are involved in biological regulation (18.8%) and cellular processes (33.9%) (Figure 5).

Meanwhile, after 14 days of extract treatments, we observed a different percentage of the proteins clustered in the classifications identified before; in only the biological process, we also revealed the modulation of the proteins involved in the metabolic process (24.3%) (Figure 6).

**Figure 7:**
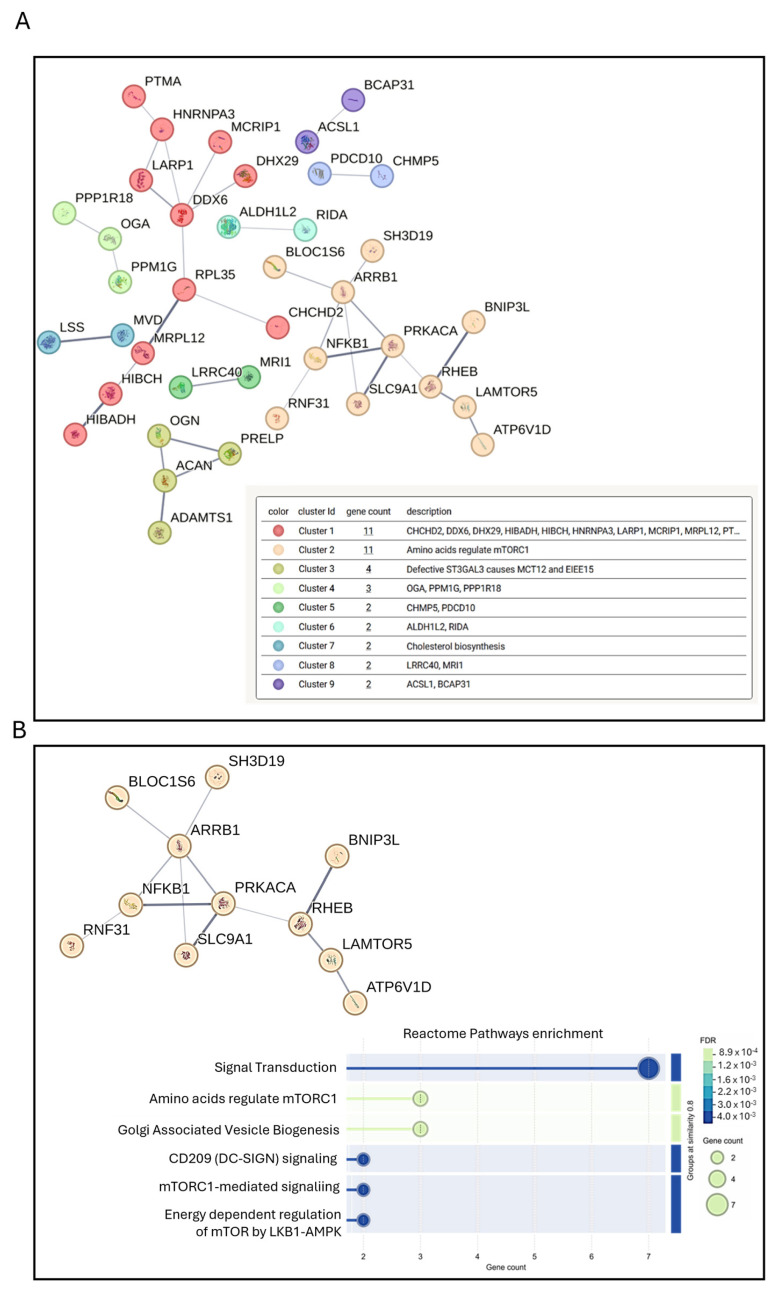
STRING protein–protein interaction analysis of secretome data. STRING K-means clusters obtained from the analysis of the proteins whose abundance was increased in the secretion from hMSCs treated with Mg AZ31+SPF+HT extracts compared to those secreted by hMSCs treated with Mg AZ32+SPF for 7 days. Proteins are indicated using their gene names. A total of nine clusters are visually defined (the names of the proteins in each cluster are reported inside the box, in the description) (**A**). Each cluster, along with the proteins it contains, is represented by a different color. (**B**) For cluster number 2, a bubble plot is used for reactome pathway enrichment analysis based on FDR (false discovery rate) and gene count.

The interaction networks for the proteins that had higher secretion levels were obtained using the STRING software. To determine which protein clusters were significantly affected by Mg AZ31+SPF+HT extracts in hMSC culture systems, STRING analysis was performed on the proteins whose secretion was higher (fold change > 1.5) for Mg AZ31+SPF+HT compared with Mg AZ31+SPF (Figure 7). After 7 days of treatment, 75 higher-abundance proteins were observed in hMSCs treated with Mg AZ31+SPF+HT compared to Mg AZ31+SPF. K-means clustering analysis revealed nine different clusters, among which the second cluster (Figure 7A) was particularly interesting. Specifically, reactome enrichment analysis demonstrated the involvement of the identified proteins in mTORC1-mediated signaling and Golgi-associated vesicle biogenesis signaling [4,40], both critical for early osteogenic differentiation (Figure 7B) [41].

**Figure 8:**
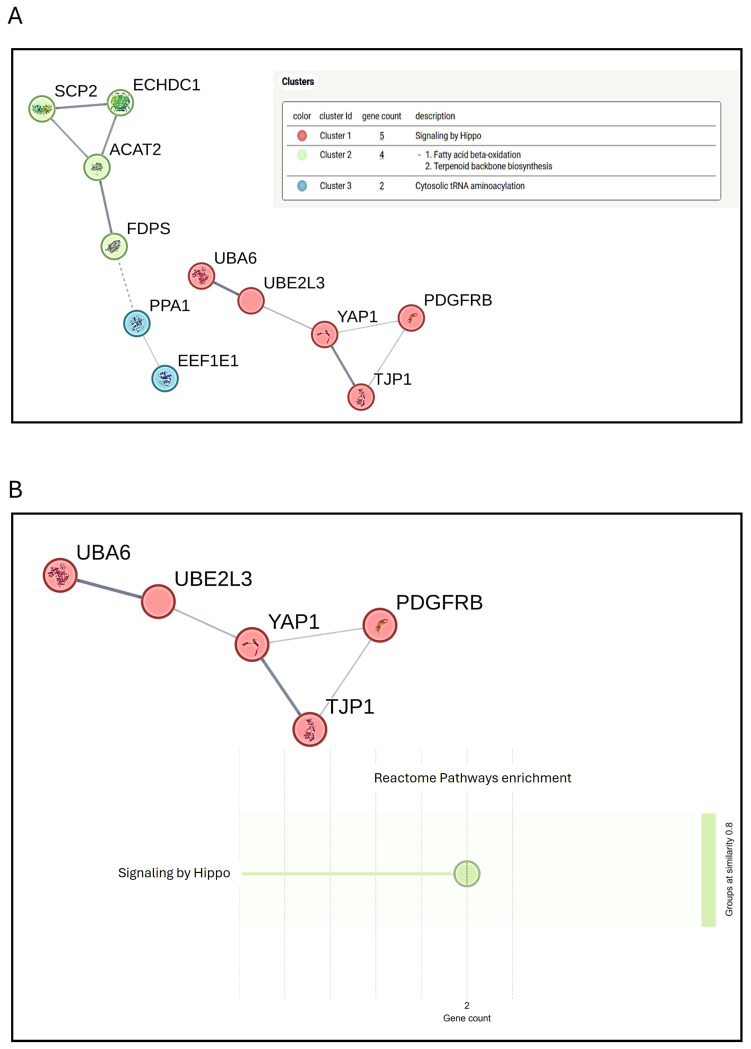
STRING protein–protein interaction analysis of secretome data. STRING k-means clusters obtained from the analysis of the proteins whose abundance was increased in the secretion from hMSCs treated with Mg AZ31+SPF+HT extracts compared to those secreted by hMSCs treated with Mg AZ32+SPF for 14 days. Proteins are indicated using their gene names. A total of three clusters are visually defined (the names of proteins in each cluster are reported in the description) (**A**). Each cluster, along with the proteins it contains, is represented by a different color. (**B**) For cluster number 1, a bubble plot is used for reactome pathway enrichment analysis based on FDR (false discovery rate) and gene count.

**Figure 9:**
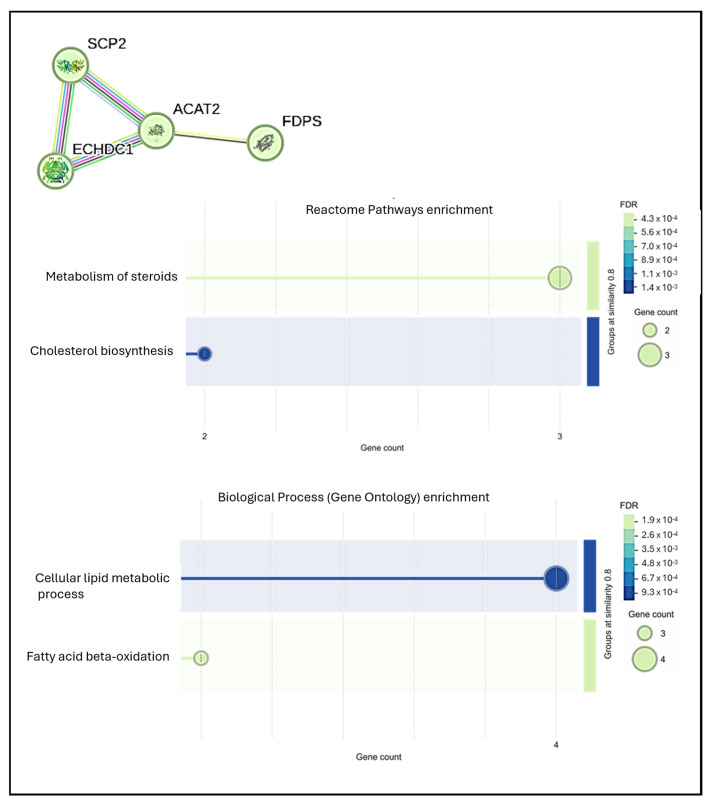
STRING protein–protein interaction analysis of secretome data. Analysis of data derived from the comparison of hMSCs treated with Mg AZ31+SPF+HT and those treated with Mg AZ31+SPF extracts after 14 days of treatments. For cluster number 2, a bubble plot is used for reactome pathway enrichment and biological process (gene ontology) enrichment analysis based on FDR (false discovery rate) and gene count.

Notably, after 14 days of treatment, 37 higher-abundance proteins were identified in hMSCs treated with Mg AZ31+SPF+HT compared to Mg AZ31+SPF (Figure 8A), most of which were related to Hyppo signaling (Figure 8B), lipid metabolism, and extracellular exosomes (Figure 9). The processes identified are strongly involved in hMSC osteogenic differentiation, triggering the metabolic changes necessary for full phenotype change.

## 4. Discussion

The quest for novel, viable materials for the creation of innovative fracture fixation systems and, indeed, prostheses has been an area of ongoing research and development for several decades. Despite the frequent introduction of new materials, current approaches remain inadequate [13]. There is an urgent need for interdisciplinary research on materials and manufacturing processes in order to achieve Mg bone implants in a clinical context [13]. In particular, surfaces are of crucial importance in regenerative medicine, as they trigger significant biological mechanisms during implantation and early bone healing [15,42]. In accordance with this need, the current study aimed to investigate the potential modifications to Mg alloy that could facilitate the overcoming of the current limitations. Mg alloys are widely regarded as biodegradable ’green’ materials as they can be synthesized from biological systems as plants, fungi, and bacteria [43,44], characterized by high strength and biocompatibility, making them very attractive in the biomedical healthcare industry. The development of novel corrosion-resistant coatings is essential for improving the corrosion resistance of magnesium alloys.

Hydrothermal synthesis is currently considered to be a highly valid method for providing a solution to this problem and for the development of in situ corrosion-resistant coatings, improving the performance of the alloys in bone regeneration research [24].

The results obtained using the in vitro hMSCs model, untreated or treated with Mg AZ31+SPF or Mg AZ31+SPF+HT extracts, revealed an enhancement in cell proliferation, as demonstrated by the increase in metabolic activity and the quantification of DNAds, as well as the notable upregulation of osteoblast genes during the experimental period. In particular, the modulation of pre-osteoblast to osteoblast gene expression occurred in a significative manner when hMSCs were treated with Mg AZ31+SPF+HT compared to Mg AZ31+SPF and cultured in OM. These findings were corroborated by a mineralization analysis performed on hMSCs maintained in OM to mimic the bone niche in vitro. Based on these results, we sought to investigate the potential involvement of Mg alloy extracts in fibrosis signaling, another crucial pathway in bone regeneration [5,42]. This is more often reported as fibrosis is a potential complication of a delayed bone regeneration process. Given the possibility that Mg alloys extracts could activate this signaling, we conducted further analysis to evaluate the effects of Mg alloy extracts on the release of pro- and anti-fibrotic proteins in hMSCs maintained in BM and OM [17,19,45]. In this study, we planned to evaluate the production of TGF-β1, TGF-β2, procollagen type I alpha1, TNF alpha, and IFN gamma in supernatants recovered from MSCs treated with Mg AZ31+SPF or Mg AZ31+SPF+HT extracts, considering the potential involvement of these factors in two key processes: successful bone tissue regeneration and fibrotic response, which impairs effective tissue repair. TGF-β signaling has widely recognized roles in bone formation, promoting MSC proliferation, the early commitment of MSCs to the osteoblast lineage, and stimulating extra cellular matrix (ECM) production [46,47,48]. On the other hand, TGF-β has been known to be a pro-fibrotic factor, and its involvement in mechanisms leading to excessive matrix synthesis is recognized [18]. Procollagen type I is the molecular precursor of type I collagen, which constitutes 90% of bone matrix proteins, so it represents a fundamental component of bone architecture and structure [49]. When uncontrolled or imbalanced synthesis and production of extracellular matrix components (such as type I collagen) occurs, overexpression of these molecules results in their excessive deposition in ECM and in fibrotic tissue formation. Finally, in order to explore potential expressions of anti-fibrotic mediators, we focused on TNF alpha and IFN gamma since their roles in controlling fibrotic response has been shown [46]. In addition, these factors are also involved in inflammatory response, which represents the first essential phase in a successful healing cascade [50]. Bioplex analysis showed that Mg AZ31+SPF+HT extracts induced an increase in the expression of anti-fibrotic and pro-fibrotic proteins compared to Mg AZ31+SPF, suggesting the ability of hydrothermal treatments of Mg AZ31+SPF to modulate one of the main osteogenic signals correlated with the osteointegration of implants. Therefore, considering the multiple roles of the investigated molecules, the results obtained by multiplex analysis performed on hMSC supernatant suggest that Mg AZ31+SPF+HT alloys, in inducing a balanced expression of pro-fibrotic/anti-fibrotic factors, seem to be able to counteract the detrimental effects of fibrotic response and to favor the beneficial functions and regenerative potential of the soluble biomolecules analyzed, promoting an osteogenic-oriented microenvironment.

The secretome of hMSCs, consisting of soluble factors (growth factors, cytokines, chemokines, and hormones) and insoluble factors contained in extracellular vesicles (EVs), plays a key role in modulating tissue regeneration through paracrine pathways [15,43]; therefore, we further monitored and compared the protein profiles of the secretome obtained from hMSCs cultured with Mg AZ31+SPF or Mg AZ31+SPF+HT alloys. Concerning this, we performed liquid chromatography with tandem mass spectrometry (LC-MS-MS) on the secretome of hMSCs cultured with Mg AZ31+SPF or Mg AZ31+SPF+HT extracts for 7 and 14 days in OM. Then, we started looking at the proteins that were upregulated when we compared the secretomes from the Mg AZ31+SPF+HT vs. the Mg AZ31+SPF culture; our interest was to identify secretory proteins that are specifically involved in the biological mechanisms of bone healing and are released after treatment with Mg extracts. The data obtained underlined the different proteins released during the experimental period. The secretome analysis performed after 7 days of treatments suggested the involvement of proteins clustered in nine processes that concerned bone formation. In particular, the K-means clustering analysis and reactome analysis underlined the release of proteins involved in m-TORC1 signaling and Golgi-associated vesicle enrichment signaling. m-TORC1 signaling is known to be related to both proliferation and osteogenic differentiation in hMSCs in vitro [51], while Golgi-associated vesicle biogenesis signaling plays a central role in bone cell functions and tissue integrity [40,41,52].

**Figure 10:**
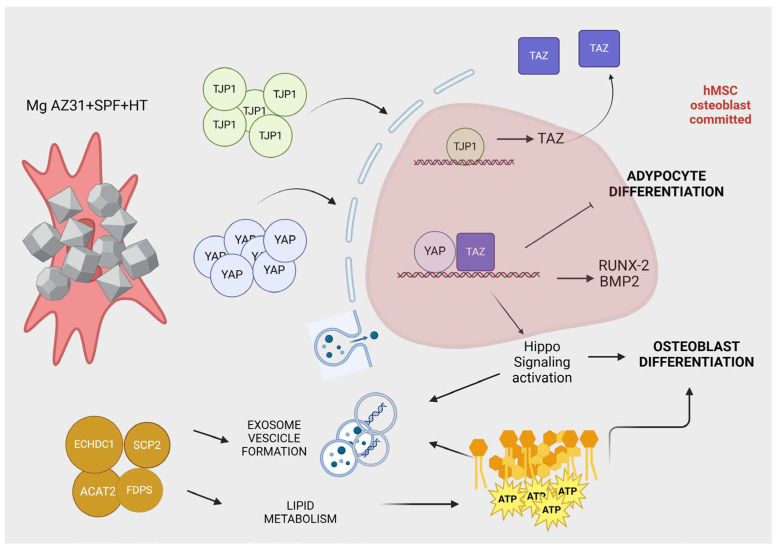
Signaling identified through secretome analysis of hMSCs treated with Mg AZ31+SPF+HT alloy extracts.

Meanwhile, a stronger modulation of proteins involved in extracellular exosomes, Hippo signaling, and lipid metabolism were observed in hMSCs after 14 days of treatment with Mg AZ31+SPF+HT extracts compared to Mg AZ31+SPF (Figure 10). Notably, these processes are involved in osteogenic differentiation, driving the hMSCs terminal differentiation. Many studies have reported the role of exosomes released from OM-treated or genetically modified hMSCs in upregulating osteogenic genes, improving mineralization nodule formation, and transporting miRNAs and lncRNAs involved in osteoblast differentiation [53]. In addition, EVs or exosomes released from the late stage of the osteogenesis of hMSCs have been shown to be involved in hydroxyapatite crystal formation [54]. Concerning these, data from the secretome underlined the different osteo-inductive effects of both Mg alloy extracts, with more emphasis placed on the pro-active effects of Mg AZ31+SPF+HT alloy compared to Mg AZ31+SPF. This suggests that these EVs could have remarkable osteogenic properties, which can be exploited for therapeutic purposes. Concerning the Hippo signaling proteins, secretome analysis revealed the increased expression of YAP when hMSCs were treated with Mg AZ31+SPF+HT extracts. YAP is a member of the YAP/TAZ complex, a transcriptional factor downstream of the Hippo pathway and a mediator of EMT signaling; of note, YAP is expressed by committed osteoblast (OB) precursors or progenitors, matrix-producing OBs, lining cells, and matrix-embedded osteocytes [46]. YAP is required to maintain cytoplasmic and nuclear pools of β-catenin, sustaining signaling activation during adult osteogenesis and bone homeostasis [55] and enhancing hMSC osteogenic differentiation by directly targeting SRY-box transcription factor 2 (SOX2) and inhibiting adipogenesis [56]. TAZ has been found to be induced by YAP expression [57] and is able to promote the osteoblast differentiation of hMSCs by increasing runt-related transcription factor 2 (Runx-2) expression and blocking adipocyte differentiation [56]. In addition, it has been demonstrated that the YAP/TAZ complex regulates macrophage polarization to promote bone defect repair and the activation of the focal adhesion kinase (FAK) signaling pathway of MSCs [57,58]. The upregulation of YAP and the STRING analysis evidence of this support the osteoinductive role of Mg AZ31+SPF+HT extracts in hMSC differentiation, in a manner that is probably synergic with extracellular exosome proteins.

STRING analysis also highlighted a lipid-related metabolism cluster, which was identified to have been upregulated after 14 days in the secretome from Mg AZ31+SPF+HT extracts when compared to Mg AZ31+SPF extracts. The upregulation of proteins involved in this cellular mechanism supported our hypothesis regarding the better osteogenic ability of Mg AZ31+SPF+HT extracts compared to Mg AZ31+SPF. Bone formation is a highly energy-demanding process. Osteoblasts have been shown to use energy from lipolysis, a process activated by lipid droplets containing endogenous fatty acids. As osteoblasts mature, different molecular pathways are activated to fuel their energy production [59]. Lipid metabolism is accelerated during hMSC osteogenic differentiation to permit the rearrangement of cytoskeletal and EV formation/delivery needed to allow phenotype changes; all these phenomena use ATP derived from glucose, ammino acids, and fatty acids. In particular, fatty acids can provide up to 40–80% of the energy produced in osteoblasts, and the alteration or modification of the genes involved in this process cause the loss of bone mass [60,61].

Overall, the evidence reported so far, although preliminary, supports the possibility that hydrothermal coatings on Mg alloys could not only overcome Mg alloy toxicity and limitations but also influence biological responses and enhance bone healing mechanisms.

## 5. Conclusions

Despite the considerable progress and numerous findings in the field of Mg alloys for medical use in bone repair in recent years, some fundamental scientific questions remain to be resolved [11,14,62]. The present study demonstrated that hydrothermal coating treatment on Mg AZ31+SPF alloy results in the formation of a protective film, which improves biocompatibility and enhances the bone regeneration process in vitro, promoting the synergistic activation of different signaling pathways involved in the osteointegration process (Figure 10). The present data highlighted that Mg AZ31+SPF+HT extracts enhanced the osteogenic differentiation of hMSCs and induced a substantial increase in mineralization nodule deposition. Notably, the secretome of hMSCs following treatment with Mg AZ31+SPF+HT was enriched with factors that may promote the initial phases of bone regeneration, which are typically initiated following prosthetic implantation. The employment of bioinformatic tools to classify protein families, their molecular functions, and their protein–protein interaction networks led to the identification of signaling mechanisms that appeared to be more involved. Among these, the Hippo and the mTOR signaling pathways were described in the context of their role in promoting bone regeneration, as evidenced by the presence of Golgi-associated vesicles. In addition, protein–protein interaction analysis revealed the enrichment of secreted proteins involved in fatty acid oxidation metabolism, which are required during osteoblast differentiation. In conclusion, the available data provide a preliminary indication of the way hydrothermal coating treatment on the Mg AZ31+SPF alloy may influence the fate of hMSCs within the niche, thereby promoting bone regeneration mechanisms. To further characterize and confirm these findings, subsequent research is planned to investigate the cellular and molecular function of the proteins identified as being important for bone regeneration.

## Figures and Tables

**Table 1:** List of Qiagen gene primers evaluated, normalized using the β-ACTIN reference gene (Table 1).

Gene	ID QuantiTect Primer Assay	Gene Globe
*ALPL*	Hs_ALPL_1_SG	QT00012957
*SP7*	Hs_SP7_1_SG	QT00213514
*BMP2*	Hs_BMP2_1_SG	QT00012544
*b-ACTIN*	Hs_ACTB_1_SG	QT00095431

## Data Availability

The datasets used and/or analyzed in the current study are available from the corresponding author upon reasonable request.
